# 2022 Acknowledgment of *mSphere Ad Hoc* Reviewers

**DOI:** 10.1128/msphere.00543-22

**Published:** 2022-12-21

**Authors:** Michael J. Imperiale

**Affiliations:** a University of Michigan Medical School, Ann Arbor, Michigan, USA

## EDITORIAL

We have a great end-of-year tradition at *mSphere*, which is taking the time to thank everyone who helps make the journal what it is. Senior editors, editors, reviewers, authors, and the American Society for Microbiology (ASM) staff all play important, complementary roles in the scientific publishing process. In 2022, we found ourselves in somewhat uncertain times as the ASM Journals program continued its transition to fully open access. Nonetheless, we are confident that *mSphere* is well positioned, having been open access since our inception, and with our impact continuing to grow each year. We are encouraged that authors have also voted with their submit buttons, so to speak, continuing to publish their excellent work with us.

The breadth of our scope, encompassing the microbial sciences writ large, means that we depend on a large and diverse pool of reviewers to guide our decisions about manuscripts. Those individuals who helped us this year are listed here and have our sincere gratitude.

Raquel Abad
Azar Abadi
Celina Monteiro Abreu
Michael C. Abt
Denise M. Akob
Kathirvel Alagesan
Bassem Allam
Rey Custer Allen
Richard A. Alm
Christopher Alteri
John Alverdy
Alfred Amambua-Ngwa
Francisco Amaro
Matthew Zack Anderson
Laura Maria Andrade de Oliveira
Alex Andrianopoulos
Linda S. Archambault
Robert A. Arkowitz
Chelsie Elizabeth Armbruster
Paul M. Arnaboldi
Gustavo Arrizabalaga
Sassan Asgari
Nick Ashbolt
Jennifer M. Auchtung
Matthew B. Avison
Steffen Backert
Anjana Badrinarayanan
Juhi Bagaitkar
Lauren O. Bakaletz
Jonathon L. Baker
Scott Baliban
Jimmy D. Ballard
Marcela Passos Galluzzi Baltazar
Jonathan M. Barasch
Roman Alfredo Barco
Brianne Barker
Samuel Barnett
Javad Barouei
Jeremy J. Barr
Stephen D. Barr
Luther A. Bartelt
Christine Marie Bassis
Andrea Battistoni
Clifford J. Beall
Jessica A. Belser
Benjamin Ross Belvin
Mar Benavides
Maureen Berg
Megan Bergkessel
Debra E. Bessen
Kyle Bibby
Kevin Bicker
Pablo Bifani
Blake Billmyre
Craig Bingman
Brian Bird
Logan Blancett
Jill R. Blankenship
Joseph M. Bliss
Patricia Pringle Bloom
Karl W. Boehme
Lydia M. Bogomolnaya
Jon Bohlin
Mélanie Bonhivers
Selina Bopp
Cristian Botta
Michael John Bottery
Teun Bousema
Kate Bowerman
Andrew S. Bowman
Allison Brady
Jonathan Braun
Michela Brazzoli
Benjamin Brennan
David J. Brenner
Christopher B. Brooke
Jeremy C. Brownlie
Emily A. Bruce
Harry Brumer
Carmen Buchrieser
Jerry M. Buysse
Matthew T. Cabeen
Edward M. Campbell
Shengbo Cao
George Carnell
Berit Carow
Jaime Carrasco
Juan Manuel Carreno
Mark Carrington
Sarah R. Carter
Fred Cassels
Marco Cassone
Roberto Cattaneo
Rodrigo Cayô
Miguel Angel Cevallos
Benjamin J. Chadwick
Chrispin Chaguza
Georgios Chamilos
Clara S. Chan
Jeffrey Chandler
Yung-Fu Chang
Muhammad Tausif Chaudhry
Huirong Chen
Liang Chen
Ye Chen
Hae Suk Cheong
Michaelle Chojnacki
Rebecca C. Christofferson
Alexander T. Ciota
Stephen A. Clark
David J. Clarke
Christine Clayton
Bryan Cobb
Shira Milo Cochavi
Darrell Cockburn
James Collins
Sean Conlan
Bede Constantinides
Laura Cook
Laura C. Cook
Isabelle Coppens
Teresa Coque
Angelina Cordone
Cynthia Nau Cornelissen
John R. Cort
Doug Cossar
Georgina Cox
Rebecca Jane Cox
Kathryn Coyne
Robert A. Cramer
Neil Crickmore
Alan Cross
Liwang Cui
Paul J. Cullen
Kathleen Cusick
Michael Cynamon
Wei Dai
Stephen Daniels
Heran Darwin
Siddhartha Das
Chandravanu Dash
Raymond James Dattwyler
Mary E. Davey
Mark R. Davies
April Dawn Davis
Zeger Debyser
Maxime Deforet
Elizabeth De Gaspari
Christopher Luis De Graffenried
Ye Deng
Nicole J. De Nisco
Jigar V. Desai
Girmay Desalegn
Santosh Dhakal
Vijaykrishna Dhanasekaran
Thomas Dick
Antonio DiGiandomenico
Francisco Dionisio
Richard D. Dix
Yohei Doi
Michael S. Donnenberg
Curtis Donskey
Charles J. Dorman
Goncalo dos Santos Correia
Zhicheng Dou
David J. Dowling
Timothy B. Doyle
Juan Du
Edward G. Dudley
Breck A. Duerkop
Joseph Alexander Duncan
Julie C. Dunning Hotopp
Frank Ebel
Leo Eberl
Adrianne N. Edwards
Andrew Edwards
Melissa Ellermann
Jeremy R. Ellermeier
Mostafa S. Elshahed
Iuliana V. Ene
Othmar G. Engelhardt
Melinda Anne Engevik
Markus Engstler
Eeva Liisa Eronen-Rasimus
Javier Antonio Escobar-Perez
José A. Escudero
Vanessa Espinosa
Morgan Evans
Christina S. Faherty
Refath Farzana
Yingang Feng
Youjun Feng
Kenneth A. Fields
D. Clark Files
Melanie J. Filiatrault
Laura Filkins
Stefan Finke
Derek J. Fisher
Fabrizio Foieni
Edward M. Fox
Michael T. France
Bettina C. Fries
Friedrich Frischknecht
Ernesto J. Fuentes
Iwona Gabriel
Starrett Gabriel
Michaela Ulrike Gack
Attila Gacser
Jennifer Gaddy
Raj Gaji
Emily Gallichotte
Ketaki Ganti
Darío García De Viedma
Meritxell Garcia-Quintanilla
Gregory Gavelis
Makda Gebre
Jennifer Geddes-Mcalister
Lee Gehrke
Lorenzo Giacani
Tim Gilberger
Steven R. Gill
Joseph James Gillespie
Susan S. Golden
Gustavo H. Goldman
Vijay Gondil
Tobias Goris
Thomas Gorochowski
Ria Goswami
Revathi Govind
Lone Gram
Timothy J. Green
Elisabeth Grohmann
Trudy H. Grossman
Sebastian Guenther
Pascale Guiton
Arthur Günzl
Shashank Gupta
Eyal Gur
Kiran Gurung
Yitzhak Hadar
Maria Hadjifrangiskou
Andrea Hahn
Mohamed A. Hakimi
Roy A. Hall
Paris S. Hamm
Jun Hang
Geoffrey D. Hannigan
Kyle Happel
Md. Manjurul Haque
Justin Hardick
Christian M. Harding
Reuben S. Harris
Ben M. Hause
Cynthia Y. He
Jianzhong He
Nicholas S. Heaton
Tory A. Hendry
Steven Hennigar
Francisca Hernandez-Hernandez
Lena J. Heung
Michael Holick
Peiying Hong
Alexander R. Horswill
Daniel K. Howe
Fupin Hu
Julian G. Hurdle
Jillian H. Hurst
Bonnie L. Hurwitz
Kevin Hybiske
Ashraf Ibrahim
Kaoru Ikuma
Iliyan Iliev
Ralph R. Isberg
Hideomi Itoh
Angela Ivask
Gulnaz T. Javan
Vicki Jeffers
Kelsea A. Jewell
Zhilong Jia
Baoming Jiang
Cheng Jin
James R. Johnson
William Johnson
Clinton J. Jones
Sheryl Justice
Björn F. C. Kafsack
Barbara C. Kahl
Jeremy Phillip Kamil
James B. Kaper
Thomas E. Kehl-Fie
Brendan Kelly
Volkhard A. J. Kempf
Lily Khadempour
M. Firoze Khan
Shahid M. Khan
Anagha Khandekar
Megan R. Kiedrowski
Nicolas Kieffer
Hye Kwon Kim
Peter E. Kima
John H. Kimbrough
Samantha Jane King
Stephen Kissler
Tomoe Kitao
Ellen Kneufer
Shintaro Kobayashi
Theresa M. Koehler
Michael H. Kogut
James B. Konopka
Anna Konovalova
Nicole Marie Koropatkin
Ioly Kotta-Loizou
Lukasz Kozubowski
Florian Krammer
Barry N. Kreiswirth
Inna V. Krieger
Erik Kristiansson
James W. Kronstad
Carol A. Kumamoto
Anand Kumar
Anuj Kumar
Mukesh Kumar
Ashlan J. Kunz Coyne
Kyohei Kuroda
Hiroyuki Kusada
Manish Kushwaha
Joseph Kusi
Olaf Kutsch
Mamuka Kvaratskhelia
Abigail L. Labella
Borden Lacy
Monique Lafon
Gyanu Lamichhane
Tilman Lamparter
Chung-Yu Lan
Kristin Lane
Stephanie N. Langel
Denis Leclerc
Allen Lee
Vincent T. Lee
Francois Le Mauff
Jose M. Lemme Dumit
Sebastian Leptihn
Cammie F. Lesser
Michael Letko
Jiasui Li
Ling Li
Yong Li
George Liechti
Efrem S. Lim
Jean Lim
Chi-Hung Lin
Yongxin Lin
Daniel Lindner
Ning Ling
Vincenzo Lionetti
Rosalia Lira
Binbin Liu
Jia Liu
Olga Lomovskaya
Jason S. Long
S. Wesley Long
Anja Lührmann
Brian Michael Luna
Liang Ma
Iain Macarthur
Antonio Machado
Matthias P. Machner
Marisa Madrid
Finlay Maguire
Andrea Maisner
Michael B. Major
Christer Malmberg
Alexander Mankin
Sam Manna
Shannon D. Manning
Nicholas J. Mantis
Kevin Maringer
Claudia N. H. Marques
Fumito Maruyama
Severine Matheus
Rosario Mato
Robin C. May
Jennifer A. Maynard
Jere W. McBride
Shonna M. McBride
Beth A. McCormick
Elizabeth A. McDaniel
Patrick McGann
Lesley McGee
Joy A. McKenna
Joseph B. McPhee
Wim G. Meijer
Jason Mercer
Timothy C. Meredith
Alita A. Miller
Kathryn C. Milligan-McClellan
Carmen Mirabelli
Dominique Missiakas
Heungyun Moon
Iain M. Morgan
James C. Morris
Karl Münger
Alison E. Murray
Mario E. Muscarella
Eleftherios Mylonakis
Joe S. Mymryk
Miki Nagao
Ryosuke Nakai
Yu Nakajima
Jadranka Nappi
Fernando Navarro-Garcia
Esther Ndungo
David R. Nelson
Martha I. Nelson
Minh Hong Nguyen
Wright W. Nichols
Helge Niemann
Leonardo Nimrichter
Laura Mary Nolan
Steven J. Norris
Fernanda Novais
Dennis Nurjadi
Joshua J. Obar
Allyson F. O'Donnell
Frank Oechslin
Kazuhiro Ogai
Toru Okamoto
Yusuke Okazaki
Daniel Groban Olson
Carlos J. Orihuela
Eva Ortega-Retuerta
Kenji Ota
Hong-Yu Ou
Brian Palenik
Glen E. Palmer
Robert J. Palmer
John C. Panepinto
Huili Pang
Hee-Soo Park
Kathryn A. Patras
Sara F. Paver
J. S. Malik S. M. Peiris
Daniel R. Perez
Vincent Perreten
Andreas Peschel
Hadrien Peyret
Catherine Ann Pfister
Jody Phelan
Gorben Peter Pijlman
Vittal Prakash Ponraj
Megan Povelones
Aditi Prabhakar
Jacob Price
Lance B. Price
Bali Pulendran
Dohun Pyeon
Leimin Qian
Alison J. Quayle
Maxime Québatte
Robert Andrew Quinn
Tara M. Randis
Tara M. Randis
Gauri G. Rao
Jayne Raper
Chad A. Rappleye
Jeremy Ratcliff
Raveen Rathnasinghe
Christina R. Rathwell
Michael L. Reese
Aaron Reinke
Justin Remais
Nilton Renno
Peter A. Rice
Dave Richard
Juergen A. Richt
James Riddell
Douglas Risser
Amariliz Rivera
Daniel D. Rockey
G. Marcela Rodriguez
Jose-Manuel Rodriguez-Martinez
Sandra Romero-Steiner
Martin Rottman
Claudia Rückert
Elizabeth Ann Rucks
Yang Rui
Thomas A. Russo
Kathrin Rychli
Robert Sabatini
Jaiprasath Sachithanandham
Marat R. Sadykov
Xavier Saelens
Mohammad Mohseni Sajadi
Davinia Salvachúa Rodriguez
Derrick Samuelson
John C. Samuelson
Nicholas D. Sanderson
Sarah E. Sansom
Panagiotis Sapountzis
Larry S. Schlesinger
Thomas M. Schmidt
Dirk Schnappinger
Michael Schuit
Stefan Schwarz
Phillip Scott
Kelly E. Seaton
Patrick R. Secor
Anna Maria Seekatz
William Self
Anna Selmecki
Chetan Seshadri
Rebecca S. Shapiro
Anupam Sharma
Ross Shaw
Lilach Sheiner
Aimee Shen
Trevor Shoemaker
Anton M. Sholukh
Erika Shor
L. David Sibley
Jeffrey Siegel
Baneshwar Singh
Ciaran Skerry
Joseph Smith
Wiep Klaas Smits
Sandro Gomes Soares
Stephanie D. Song
Joseph A. Sorg
Shanmuga Sozhamannan
Jennifer K. Spinler
Stanley M. Spinola
Shiranee Sriskandan
Richard Stanton
Gabriel J. Starrett
Chad Steele
David S. Stephens
Wolfgang R. Streit
Mikael Lenz Strube
Xin-Zhuan Su
Karthik Subramanian
Yo Sugawara
Daigo Sumi
Nobuhiro Suzuki
Yasuhiro Suzuki
Mary Hannah Swaney
Jason B. Sylvan
Clifford C. Taggart
Akifumi Takaori-Kondo
Michal Caspi Tal
Rita Tamayo
Satoshi Tamazawa
Ying Taur
Sam R. Telford
Lesly A. Temesvari
Sharon Mei Tennant
Benno Herman Ter Kuile
Rita Tewari
Elitza S. Theel
Kevin R. Theis
Torsten Thomas
Cecilia Thompson
Yun Tian
Maria Tomas
Dieter M. Tourlousse
Masanori Toyofuku
Katrina E. Traber
John S. Tregoning
Andrea Trevisan
David R. Tribble
David Turra
Jane F. Turton
Yuki Uehara
Svetlana Ugarcina Perovic
Elizabeth R. Unger
Jane Usher
Francisco Uzal
Krystal Vail
Christiaan van Ooij
Jan-Peter van Pijkeren
Caroline Elisabeth Visser
Romain Volle
Jay Vornhagen
Sarah Vreugde
Willem Waegeman
Seth T. Walk
David H. Walker
David A. Walsh
Shanquan Wang
Chongzhen Wang
Luxin Wang
Tian Wang
Tiffany Weinkopff
David S. Weiss
Louis M. Weiss
Volkmar Weissig
Stephen Robert Welch
Kristen E. Wendt
Eric Wenzler
Edze Westra
Dawn Wetzel
Annegret Wilde
Michael R. Wiley
Mary E. Wilson
Steven S. Witkin
Kenneth H. Wolfe
Manuel Wolters
Patrick C. Y. Woo
R. Mark Wooten
Floyd L. Wormley, Jr.
Karen L. Wozniak
Rachel A. F. Wozniak
Xianfu Wu
Xin Xu
Chaoyang Xue
Tetsuo Yamaguchi
Kyosuke Yamamoto
Dong Yang
Yiling Yang
Ozlem Yilmaz
Hyunah Yoon
Mikaeel Young
Jae-Hyuk Yu
Yunsong Yu
Joseph P. Zackular
María Mercedes Zambrano
Merve S. Zeden
Jin Zeng
Bing Zhai
Kai Zhang
Shengda Zhang
Wei Zhang
Weiping Zhang
Zhaolei Zhang
Jiangchao Zhao
Xian-Liang Zhao
Xilin Zhao
Chunfu Zheng
Haixue Zheng
Qingfei Zheng
Xiaoxian Zhong
Panpan Zhou
Erwin G. Zoetendal

